# P-1473. Amoxicillin-Clavulanate Transition Therapy in ESBL Urinary Tract Infections

**DOI:** 10.1093/ofid/ofae631.1643

**Published:** 2025-01-29

**Authors:** Abigail Radoncic, Maggie Box

**Affiliations:** Scripps Health, San Diego, California; Scripps Memorial Hospital La Jolla, La Jolla, California

## Abstract

**Background:**

Extended-spectrum beta-lactamase (ESBL)-producing bacteria are notoriously resistant to many antibiotics, resulting in limited treatment options for such infections. Pathogens that possess this resistance mechanism typically necessitate the use of broad-spectrum antibiotics. An often-overlooked option for ESBL urinary tract infections (UTIs) is amoxicillin-clavulanate (AMC). Currently, there is a dearth of evidence supporting its use. This analysis was done to assess the efficacy of AMC on ESBL UTI clinical cure and associated outcomes.

**Methods:**

This was a retrospective chart review conducted at Scripps Memorial Hospital La Jolla between January 1^st^, 2018 and January 31^st^, 2023. Adults with UTIs caused by ESBL pathogens who completed an AMC course as UTI treatment were included. The primary outcome was clinical cure, defined by the absence of reported symptoms in hospital or clinic encounters within 7 days of AMC completion. Secondary outcomes included 30-day relapse (UTI with the same organism within 30 days), 30-day reinfection (UTI with a different organism within 30 days), or 90-day recurrence (infection with same organism within 90 days).

**Results:**

A total of 196 patients were screened for enrollment, of which 100 were included. Uncomplicated cystitis (UC) was identified in 39% of patients, complicated cystitis (CC) in 17%, pyelonephritis in 25%, and catheter-associated UTI (CAUTI) in 19%. Concomitant bacteremia was identified in 9% of patients. AMC oral transition (IV step down) therapy was used in 61% of patients. Overall, 7-day clinical cure regardless UTI type was 96%. Clinical cure was 97.4% for UC, 94.1% for CC, 92% for pyelonephritis, and 100% for CAUTI. Of the patients who received AMC as primary therapy (n = 39), 100% achieved 7-day clinical cure. Clinical cure in those with an AMC MIC of ≤ 4/2 was 95.8% (n =56) and in those with a MIC of 8/4 was 96.2% (n = 53). In those with concomitant bacteremia, 77.8% (7/9) achieved 7-day clinical cure. UTI 30-day relapse, 30-day reinfection, and 90-day recurrence occurred in 17%, 8%, and 16% of patients, respectively.

**Conclusion:**

Amoxicillin-clavulanate may be a promising oral transition therapy for ESBL UTIs. Larger, more robust studies should be done to analyze the efficacy of AMC as an option for primary or transition therapy for ESBL UTIs.

**Disclosures:**

**All Authors**: No reported disclosures

